# *Drosophila* TRPM Channel Is Essential for the Control of Extracellular Magnesium Levels

**DOI:** 10.1371/journal.pone.0010519

**Published:** 2010-05-06

**Authors:** Thomas Hofmann, Vladimir Chubanov, Xiaodi Chen, Anna S. Dietz, Thomas Gudermann, Craig Montell

**Affiliations:** 1 Institut für Pharmakologie und Toxikologie, Philipps-Universität Marburg, Marburg, Germany; 2 Walther-Straub-Institut für Pharmakologie und Toxikologie, Ludwig-Maximilians-Universität, München, Germany; 3 Departments of Biological Chemistry and Neuroscience, Center for Sensory Biology, Johns Hopkins University School of Medicine, Baltimore, Maryland, United States of America; University of Texas MD Anderson Cancer Center, United States of America

## Abstract

The TRPM group of cation channels plays diverse roles ranging from sensory signaling to Mg^2+^ homeostasis. In most metazoan organisms the TRPM subfamily is comprised of multiple members, including eight in humans. However, the *Drosophila* TRPM subfamily is unusual in that it consists of a single member. Currently, the functional requirements for this channel have not been reported. Here, we found that the *Drosophila* TRPM protein was expressed in the fly counterpart of mammalian kidneys, the Malpighian tubules, which function in the removal of electrolytes and toxic components from the hemolymph. We generated mutations in *trpm* and found that this resulted in shortening of the Malpighian tubules. In contrast to all other *Drosophila trp* mutations, loss of *trpm* was essential for viability, as *trpm* mutations resulted in pupal lethality. Supplementation of the diet with a high concentration of Mg^2+^ exacerbated the phenotype, resulting in growth arrest during the larval period. Feeding high Mg^2+^ also resulted in elevated Mg^2+^ in the hemolymph, but had relatively little effect on cellular Mg^2+^. We conclude that loss of *Drosophila trpm* leads to hypermagnesemia due to a defect in removal of Mg^2+^ from the hemolymph. These data provide the first evidence for a role for a *Drosophila* TRP channel in Mg^2+^ homeostasis, and underscore a broad and evolutionarily conserved role for TRPM channels in Mg^2+^ homeostasis.

## Introduction

Cellular Mg^2+^ levels must be tightly controlled to ensure the appropriate activities of a host of enzymes and other proteins critical for metabolism, neuronal excitability and muscle contraction [1]. Mg^2+^ homeostasis is accomplished in mammals through a balance between absorption of ingested Mg^2+^ by the intestine, filtration and reabsorption of excessive Mg^2+^ by the kidneys and storage in the bone [1], [2], [3], [4].

A variety of transporters and channels are critical for Mg^2+^ homeostasis, including members of the TRP superfamily of cation channels [5], [6], [7]. These include several TRPM channels, such as TRPM7 and the highly related channel TRPM6. Loss of TRPM7 in cultured chicken DT40 B-lymphocytes causes lethality and this phenotype is reduced by supplementation of the media with high levels of Mg^2+^ [8], although a direct effect on Mg^2+^ homeostasis does not appear to explain the developmental phenotype in TRPM7 deficient mouse thymocytes [9]. TRPM6 is expressed in the intestines and kidneys and mutations in this Mg^2+^ and Ca^2+^ permeable channel lead to low serum levels of Mg^2+^ and Ca^2+^ [10], [11]. The hypomagnesemia with secondary hypocalcemia (HSH) result in neurological problems, including seizures, which can be ameliorated by high doses of dietary Mg^2+^ [10], [11]. Thus, TRPM6 appears to be essential for Mg^2+^ absorption in the intestines. None of the six other mammalian TRPM channels have been associated with Mg^2+^ homeostasis. Rather, they participate in a variety of other functions such as taste transduction [12], [13] and the sensation of cool temperatures [14], [15]. The worm, *C. elegans*, encodes three TRPM channels, two of which GON-2 and GTL-1 are expressed in the intestines and function in Mg^2+^ homeostasis [16]. Worms that are mutant for both *gon-2* and *gtl-1* display a growth defect, which is suppressed by supplementation of the food with high Mg^2+^ [16]. A third worm TRPM channel appears to function in Mg^2+^ homeostasis in the excretory cell [17]. *Drosophila* encodes a single TRPM protein; however, the physiological role of this protein has not been described. Unlike mammalian TRPM2 and TRPM6/7 [6], the fly TRPM protein does not include a linked enzyme domain.

Here we report the generation and characterization of mutations affecting the *Drosophila trpm* gene, which is expressed in the functional equivalent of mammalian kidneys, the Malpighian tubules. The *trpm* mutant larvae were viable, but developed more slowly than wild-type. The animals exhibited morphological defects in the Malpighian tubules and underwent complete developmental arrest as prepupae. The *trpm*-deficient larvae were extremely sensitive to elevated dietary Mg^2+^ levels as these feeding conditions led to a pronounced hypermagnesemia. These data suggest that *Drosophila trpm* mutants develop hypermagnesemia due to impairment in homeostatic removal Mg^2+^ from the hemolymph by the Malpighian tubules.

## Results

### Loss of *trpm* lead to larval growth retardation and pupal arrest

**Figure 1:**
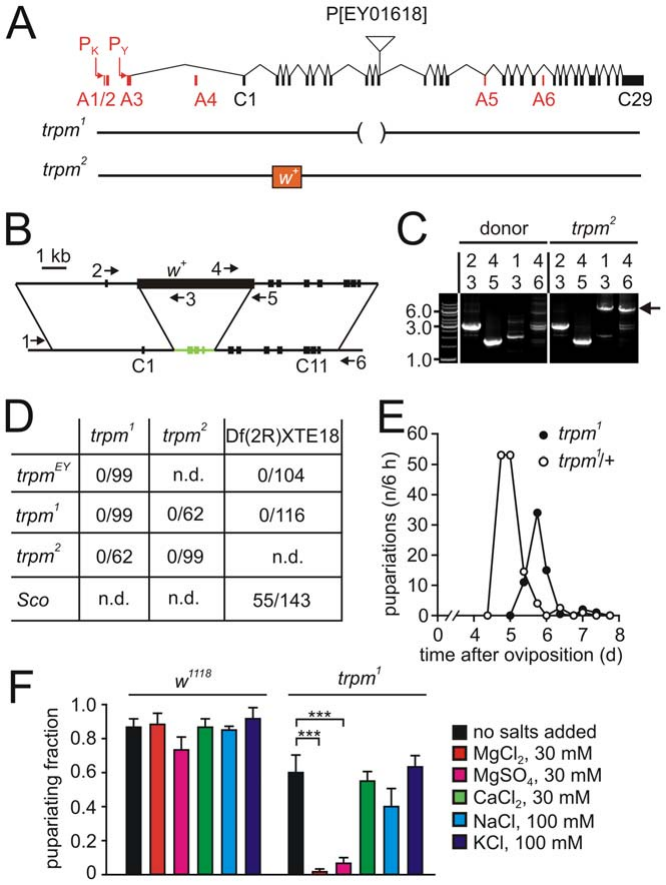
Requirement for *trpm* for viability. (A) Intron-exon structure of the *trpm* gene and generation of *trpm* alleles. Exons conserved in all splice variants are in black (C1-29) and alternative exons (A1–A6) as well as the two alternative transcription starts P_K_ and P_Y_ are in red. Shown below are the modifications in the *trpm^1^* and *trpm^2^* alleles. The *trpm^1^* allele is missing C9-11 as a result of imprecise excision of the P element line P[EY01618]. The *trpm^2^* allele contains a *w^+^* marker in place of exons C2–C4. (B) PCR primers (indicated by the numbered arrows) used for confirming the deletion in *trpm^2^*. (C) PCR analyses of genomic DNA from the donor transgenic flies and following successful homologous recombination (*trpm^2^*). Primers 3 and 4 corresponded to sequences within the *w^+^* reporter gene. Primers 2 and 5 were complementary to sequences in the donor construct and primers 1 and 6 corresponded to DNA in the *trpm^2^* gene but not in the donor transgene. Consequently the 2/3 and 4/5 primer pairs produced identical PCR products using DNA from either donor or *trpm^2^* flies while the 1/3 and 4/6 primer pairs produced products using *trpm^2^* DNA only. (D) The *trpm* mutations prevent survival to adulthood. The number of adult flies produced following the crosses between the indicated flies. All of the indicated mutations and the deficiency spanning the *trpm* locus were placed *in trans* with the CyO balancer. The P-element line EY01618 is indicated as *trpm^EY^*. The numerator indicates the number of adults without the balancer and the denominator indicates the number of adults with the indicated chromosome *in trans* with CyO. (E) Growth delay in the *trpm^1^* mutant. The number of days required for formation of pupae. Pupae were scored every 6–12 h. (F) Reduction of the fraction of *trpm^1^* larvae reaching pupariation within 7 days due to the ions supplemented in the food as indicated. The *w^1118^* strain was used as the *trpm^+^* control.

Most metazoan organisms that have been subjected to genome-wide sequence analysis encode multiple TRPM channels [6]. These include eight in humans and mice and three in worms (Figure S1A). In contrast, insects such as fruit flies, bees and mosquitoes encode one TRPM isoform. Thus, mutation of the single *trpm* gene in an insect eliminates all TRPM channel function. To characterize the physiological requirements for TRPM channels, we focused on the *Drosophila trpm* locus (*CG34123*). We found that the *trpm* gene was expressed as an array of alternative spliced isoforms, four of which encode proteins of 1909 to 2022 residues (Figure S1B). These predicted TRPM proteins are most similar to mammalian TRPM1 and TRPM3. The RNAs all included 29 common exons (C1 – 29; Figure 1A and Figure S1B). The *trpm* RNA was expressed at various developmental stages at the expected size (∼6 kb; Figure S1C). Due to the low expression, it was not feasible to probe Northern blots with isoform-specific probes.

To characterize the requirements for *trpm in vivo*, we generated a deletion affecting the *trpm* gene. We mobilized a P-element, P[EY01618], which inserted in the 3′ splice site of exon C11 (Figure 1A), and identified a line (*trpm^1^*) with an imprecise excision that removed three exons (C9 – C11) encoding residues 364 to 511. To generate a second independent *trpm* allele, we used ends-out homologous recombination [18] to insert the *w^+^* gene in place of exons C2 – C4 (*trpm^2^*; residues 38 to 162; Figures 1A–1C). The deletions in both alleles also changed the reading frame.

The *trpm^1^* or *trpm^2^* mutations resulted in pupal lethality. Lethality also resulted when we placed either the *trpm^1^* or *trpm^2^* chromosomes *in trans* with a deficiency, Df(2R)XTE18, which uncovered the locus (Figure 1D). The predicted numbers of homozygous *trpm^1^* embryos were produced, and the 1^st^ instar larvae hatched at a similar time as the wild-type and heterozygous controls (data not shown). Furthermore, the numbers of 3^rd^ instar *trpm^1^* larvae that reached pupariation were not reduced relative to heterozygous controls from the same egg collection. However, the time-to-pupariation was delayed ∼1 day (Figure 1D). Since larvae were produced and no flies eclosed from the pupal cases, we conclude that loss of *trpm* resulted in pupal lethality.

### Sensitivity of *trpm^1^* animals to high Mg^2+^ levels

**Figure 2:**
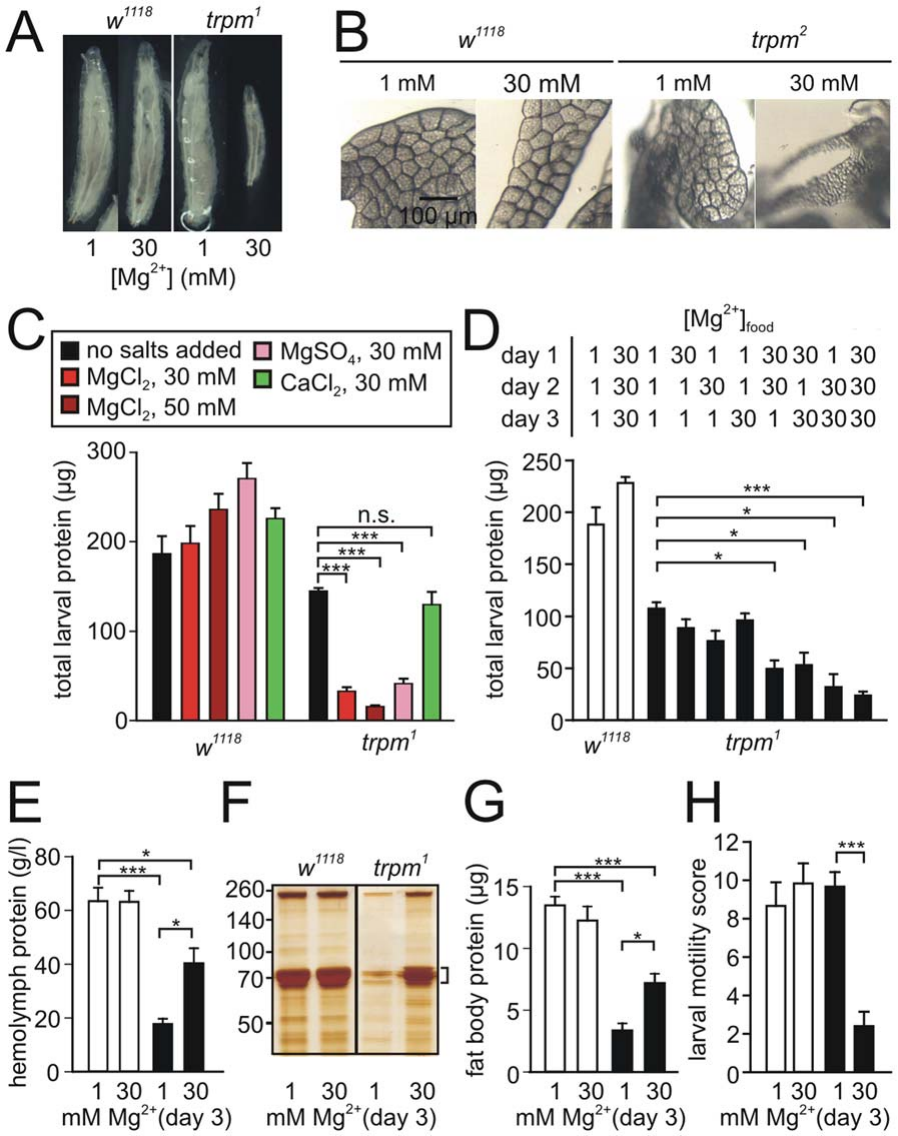
Impact of dietary Mg^2+^ on larval growth in the *trpm* mutant. (A) Sizes of larvae after feeding for three days on food containing 1 or 30 mM total Mg^2+^. (B) Fat bodies dissected from *w^1118^* and *trpm^2^* 3^rd^ instar larvae fed on either 1 mM or 30 mM Mg^2+^. (C) Summary of total larval protein per larvae after 80 hrs on media supplemented with the indicated divalent cation salts (n = 5−10). (D) Sensitivity of *trpm* larvae (*trpm^1^*) to the duration of Mg^2+^ exposure. Larvae were kept in media containing 1 or 30 mM MgCl_2_ for 1–3 days as indicated. (E) Hemolymph protein content in larvae exposed to low and high levels of MgCl_2_ (white bars, Canton S; black bars, *trpm^2^*). (F) Detection of hemolymph proteins by silver staining after by SDS-PAGE fractionation of the equivalent of 160 nl of hemolymph in each lane. Protein sizes markers are indicated to the left (kDa) and the bracket indicates the LSP proteins/hexamerins. (G) Protein content in the fat bodies microdissected from 3^rd^ instar larvae after being maintained on day 3 on either 1 or 30 mM Mg^2+^. (H) Effect of 1 day of exposure to elevated Mg^2+^ on the locomotive performance of *w^1118^* and *trpm^1^* larvae. 3^rd^ instar larvae were allowed to move freely on a 2% agar field demarcated with a projected 1 cm grid. We then assayed the number of lines crossed after 3 min (n = 5−6 each, mean +/− SEM). The level of statistical significance of the difference between groups is indicated above the bars (*, p<0.05; ***, p<0.001, n.s. not significant).

Given that members of the TRP superfamily are cation channels, some of which are known to be essential for divalent cation homeostasis, we wondered whether the *trpm* pupal lethality might be enhanced or suppressed by supplementing the food with increased levels of cations. We found that addition of 30 mM Mg^2+^ to the standard food (1 mM Mg^2+^) caused nearly complete larval lethality, as no homozygous larvae reached the pupal stage (Figure 1F). In contrast to these results, high Mg^2+^ had no effect on survival of *trpm^+^* control animals (*w^1118^*; Figure 1F). Introduction of a variety of other cations (CaCl_2_, NaCl and KCl) to the food had no effect on larval survival of *trpm^1^* or the rate of wild-type development (Figure 1F). When we added 10–30 mM MgCl_2_ to the food, the larvae were smaller and thinner than wild-type (Figure 2A), and the cells in the fat bodies were dramatically reduced in size (Figure 2B and Figure S2). However, when we reared the larvae on standard food (0.5–1 mM Mg^2+^), the overall dimensions of the *trpm^1^* larvae and the size of the fat body cells were reduced only slightly relative to wild-type (Figure 2A and B).

Consistent with the smaller size of *trpm* mutant larvae that were fed high levels of Mg^2+^, we found that the total amount of protein per larvae was reduced greatly. Mutant larvae reared on standard media with no added salts (1 mM Mg^2+^) displayed only a relatively modest, although statistically significant reduction in protein content (Figure 2C). However, the protein content was dramatically decreased in those larvae reared on high concentrations of Mg^2+^ (Figure 2C). This effect was independent of the specific anion (Cl- or SO_4_^2−^) and was not mimicked by a similarly high concentration of Ca^2+^ (Figure 2C).

We wondered whether there was a critical period during larval growth that was more sensitive to the inhibitory effects of high Mg^2+^. To conduct this analysis we switched the larvae between food containing either 1 mM or 30 mM Mg^2+^ at various times during the first three days of larval growth and measured total larval protein. The protein mass in wild-type larvae was unchanged regardless of whether they were reared with food containing the high or low Mg^2+^ levels for the first three days of larval development (Figure 2D). If the *trpm^1^* larvae were maintained on 30 mM Mg^2+^ during day one or two (1^st^ and 2^nd^ instar larvae, respectively), there was a relatively mild decrease in protein content, and exposure to high Mg^2+^ during day three only had little effect (Figure 2D). However, if the larvae were fed high Mg^2+^ for two days the deleterious effect was intermediate between those fed high Mg^2+^ for 1 or 3 days (Figure 2D).

The preceding data indicate that the effect of Mg^2+^ was proportional to the length of exposure to this diet. However, there was one notable exception. If we supplemented the food with high Mg^2+^ on day 3 only (early 3^rd^ instar larvae) there was no significant reduction in total larval protein in the *trpm^1^* animals, relative to the mutant larvae maintained on normal (1 mM Mg^2+^) food (Figure 2D).

Many of the proteins in the hemolymph are synthesized and secreted by the fat bodies. Most abundant of these proteins are the hexamerins (larval serum proteins, LSPs 1 and 2 [19]. We found that in mutant larvae fed constantly on standard food (1 mM Mg^2+^), the total protein content in the fat bodies were reduced relative to wild-type (Figure 2E), and the concentration of proteins in the hemolymph, including the LSPs, were decreased greatly (Figure 2F and G). However, if 30 mM Mg^2+^ were fed to the *trpm^1^* early 3^rd^ instar larvae (on day 3), the total protein content in the fat bodies and hemolymph, and the levels of secreted LSPs were increased significantly (Figure 2E–G). On the other hand, exposure of *trpm^1^* larvae to elevated Mg^2+^ for one day (day 3) resulted in a marked reduction in locomotor activity (Figure 2H), along with a reduction in foraging activity (data not shown). Thus, loss of *trpm* caused phenotypes that were enhanced or suppressed by high Mg^2+^, and their relative impact on overall growth depending on the developmental stage in which the larvae were exposed to the Mg^2+^ supplementation.

### Hypermagnesemia in *trpm* larvae

**Figure 3:**
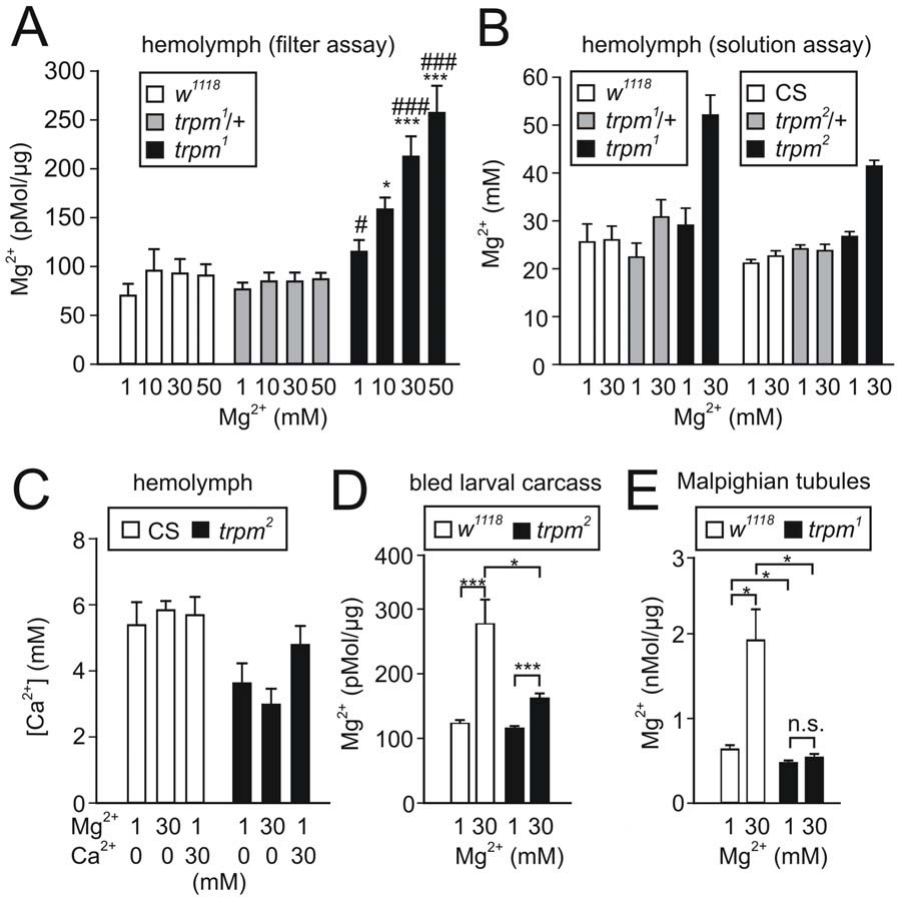
Hypermagnesemia due high dietary Mg^2+^. The quantification of Mg^2+^ and Ca^2+^ concentrations were determined after exposing early 3^rd^ instar stage larvae to the indicated concentrations of dietary Mg^2+^ for 24 h. (A) Mg^2+^ content in hemolymph from individual larvae exsanguinated on Whatman 3 MM paper. The Mg^2+^ content was normalized to the protein content of the larval carcass. (B) Mg^2+^ concentration in hemolymph collected into capillary tubes. Each sample tested consisted of hemolymph pooled from 8–10 larvae each from the indicated genotypes (left set of data, n = 3 pools each; right set of data, n = 5 pools each). CS, Canton S strain of wlld-type flies. (C) Hemolymph Ca^2+^ concentrations determined using arsenazo III. The assays were performed with the same hemolymph pools from right set of panel (B). (D) Mg^2+^ retained in the bled carcasses of *trpm^2^* larvae. (E) Mg^2+^ in the Malpighian tubules. The levels of Mg^2+^ were normalized to the protein content. Both pairs of Malpighian tubules, with a small portion of gut remaining were microdissected, homogenized and subjected to Mg^2+^ determination and protein quantification (n = 24−27 tubule sets each). Error bars represent SEMs.

Flies and larvae contain an open circulatory system so that the functional equivalent of mammalian blood (hemolymph) baths the internal tissues. We determined the effects of high Mg^2+^ supplementation on the levels of Mg^2+^ in the hemolymph using two approaches. To use similarly sized wild-type and *trpm^1^* larvae, we reared the larvae on standard food (1 mM Mg^2+^) for two days, before switching the animals to 10–50 mM Mg^2+^ for the third day. In the first approach, we bled out the hemolymph onto filter paper and normalized the concentration of Mg^2+^ to the protein mass in the remaining carcasses. Using this filter assay, the concentration of Mg^2+^ in the wild-type hemolymph was similar regardless of whether they were fed on diets containing the low or high Mg^2+^ concentrations (Figure 3A). When *trpm* mutant larvae were reared on the 1 mM Mg^2+^, the level of Mg^2+^ in the hemolymph was indistinguishable from wild-type (Figure 3A). Of significance here, the concentration of Mg^2+^ in the hemolymph approximately doubled when the mutant animals were fed high mM Mg^2+^ (Figure 3A). We also collected hemolymph and measured the Mg^2+^ levels in solution. Once again, we found that the Mg^2+^ hemolymph levels increased specifically in the mutant when high levels of Mg^2+^ were added to the food (Figure 3B). However, high or low Mg^2+^ had no impact on the hemolymph Mg^2+^ concentration in either of the wild-type control strains (Canton S or *w^1118^*).

We also assayed the levels of Ca^2+^ in the hemolymph, using the arsenazo III complexing agent. We found that the Ca^2+^ levels same in wild-type larvae feed high or low Mg^2+^ or Ca^2+^ (Figure 3C). However, the hemolymph Ca^2+^ levels were reduced slightly in *trpm* mutant larvae regardless of the concentration of Mg^2+^ or Ca^2+^ in the food (Figure 3C)

### Failure of *trpm* Malpighian tubules to concentrate Mg^2+^

To determine whether feeding a 30 mM Mg^2+^ diet also caused an elevation in the concentration of Mg^2+^ in tissues, we quantified Mg^2+^ levels in larval carcasses after we bled out the hemolymph. We found that if the larvae were fed 1 mM Mg^2+^, the concentrations of Mg^2+^ were similar in the wild-type and *trpm* carcasses (Figure 3D). After consuming 30 mM Mg^2+^ for 24 hours, the tissue Mg^2+^ level in wild-type larvae was elevated greatly (Figure 3D). However, under the same feeding conditions, there was only a slight increase in the concentration of Mg^2+^ in the *trpm* tissues (Figure 3D).

In *Drosophila*, the initial and transitional segments of the Malpighian tubules are major sites for intermediate storage of Mg^2+^ and Ca^2+^ ions, which occur in the form of intracellular and intraluminal concretions [20]. This region of the Malpighian tubules also contributes to homeostatic Mg^2+^ excretion [20]. Therefore, we examined the relative levels of Mg^2+^ in wild-type and *trpm* mutant Malpighian tubules and normalized the measurements based on total protein content. In wild-type animals the Mg^2+^ content was greatly elevated after they were placed on the diet containing 30 mM Mg^2+^ (Figure 3E). Since this concentration was four to five-fold higher than the Mg^2+^ in total tissues, this result was consistent with increased intraluminal storage of Mg^2+^ under these conditions. In contrast, the level of Mg^2+^ in the *trpm* Malpighian tubules was unaffected by the concentration of Mg^2+^ in the diet (Figure 3E). The lack of increase in Mg^2+^ concentration in the Malpighian tubules, combined with the higher levels of Mg^2+^ in the hemolymph suggest a defect in Mg^2+^ uptake in this tissue.

### TRPM is expressed in and affects the size of Malpighian tubules

**Figure 4:**
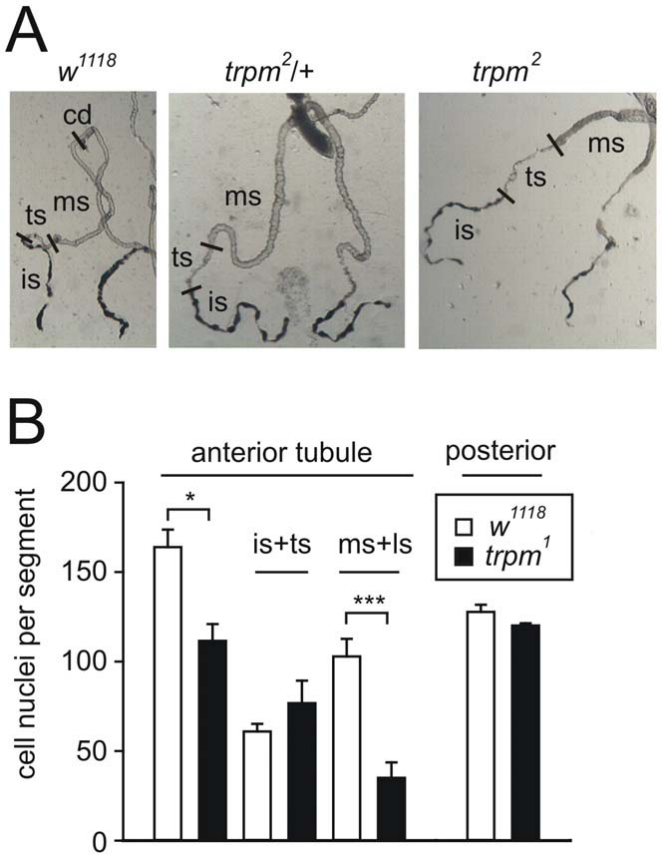
Altered Malpighian tubule morphology in the *trpm* mutant. (A) Representative images of anterior tubule pairs microdissected from 3^rd^ instar larvae. The approximate location of the different segment borders are indicated: cd  =  collecting duct, ms  =  main segment, ts  =  transitional segment, is  =  initial segment). (B) Cell numbers in the *w^1118^* and *trpm* tubules. The tubules were fixed and stained with DAPI and the numbers of nuclei in each tubule/segment were tabulated. Error bars represent SEMs.

We found that the length and morphology of the larval Malpighian tubules was affected in the *trpm* 3^rd^ instar larvae. The anterior tubule pair was 30% shorter than wild-type and contained fewer cells (Figure 4A and B). This difference was restricted to the main segment and the lower segment, while the transitional segment, initial segment and posterior tubules were similar in length to wild-type (Figure 4A and B).

**Figure 5:**
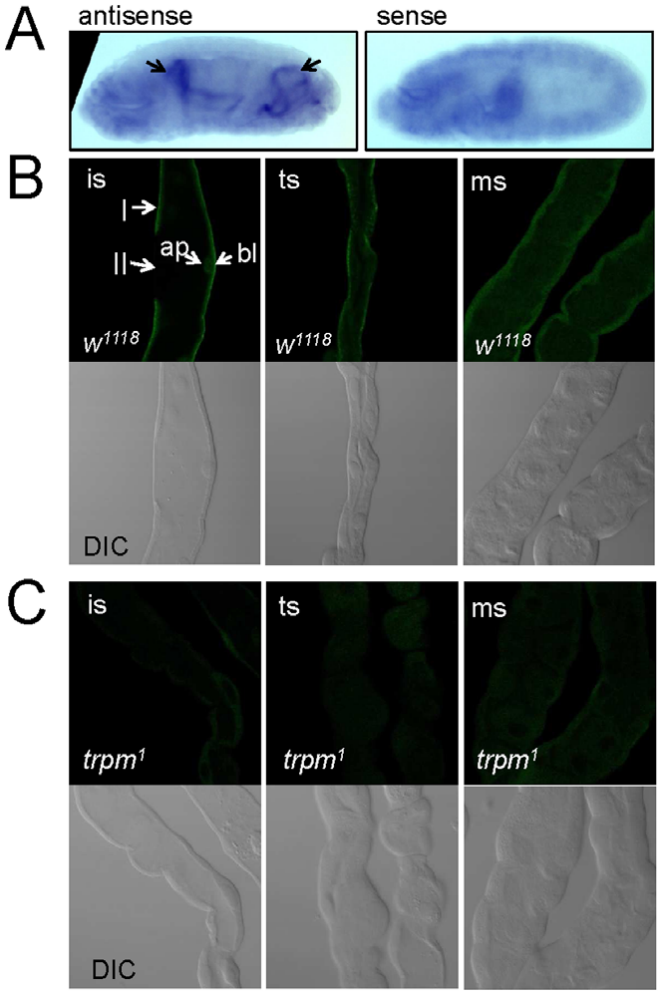
Expression of *trpm* in Malpighian tubules. (A) Detection of *trpm* RNA in Malpighian tubules of stage 16–17 embryos by *in situ* hybridization. Arrows indicate the localization of the anterior and posterior Malpighian tubules in the left panel using the antisense probe. (B) Malpighian tubules from *w^1118^* 3^rd^ instar larvae stained with TRPM antibodies. (C) Malpighian tubules from *trpm^1^* 3^rd^ instar larvae stained with TRPM antibodies. Abbreviations: ms = main segment, ts = transitional segment, is =  initial segment, I = type 1/principal cell, II =  type 2/stellate/bar-shaped cell. The stainings of the epithelial apical (ap) and basolateral compartments are indicated.

To determine whether *trpm* was expressed in Malpighian tubules, we examined the expression pattern of the RNAs and proteins. To characterize the spatial distribution of the *trpm* RNA, we performed *in situ* hybridizations using late stage embryos. We found that the *trpm* signal labeled the Malpighian tubules most prominently (Figure 5A). While some signal appeared in variable regions of the embryo with the sense control, in contrast to the anti-sense probe, the control did not label any specific region consistently and never stained the Malpighian tubules.

To examine expression of the TRPM proteins in Malpighian tubules we prepared TRPM antibodies. Although the antibodies were effective on Western blots containing extracts from tissue culture cells expressing TRPM, they did not recognize TRPM on Western blots using extracts form native tissue (data not shown), possibly due to low expression levels. Nevertheless, we detected anti-TRPM signals in the Malpighian tubules of wild-type but not mutant 3^rd^ instar larvae (Figure 5B and C). The signals were in the basolateral portions of the initial and transitional segments of anterior tubules (Figure 5B), which are the segments thought to function in excretion of divalent cations. The initial segment includes stellate cells (sc; type 2 cells) and principal cells (pc; type 1 cells) [21]. We found that the TRPM antibodies stained the principal cells, but not the stellate cells. Thus, TRPM was expressed in the regions of the Malpighian tubules that function in storage of intraluminal Mg^2+^ concretions and Mg^2+^ secretion [20].

## Discussion

The Malpighian tubules, which represent the *Drosophila* functional equivalent of mammalian kidneys, have been studied extensively as a model for renal function and epithelial fluid transport [22]. Several ion channels, including the IP_3_-receptor and the TRPL channel have been shown to participate in fluid transport in Malpighian in response to capa family neuropeptides [23], [24]. However, the *Drosophila* channels required for electrolyte homeostasis are not known.

Several observations in the current report indicate that TRPM functions in Mg^2+^ homeostasis. The TRPM protein is expressed in the principal cells of the Malpighian tubules, which are necessary for filtering electrolytes from the hemolymph and for excretion, and *trpm* mutations impair the morphology of the Malpighian tubules. Of primary importance in this study, the *trpm* animals were unable to handle high levels of Mg^2+^ in the diet. Supplementation with 30 mM Mg^2+^ led to a pronounced elevation of Mg^2+^ in the hemolymph, and a growth arrest during the larval period. The combination of these data indicated that loss of *trpm* resulted in altered Malpighian tubules, leading to impaired excretion of excessive Mg^2+^. Although we were unable to perform rescue experiments with a wild-type transgene, due to the large genomic region and the wide array of mRNA isoforms, the phenotypes described here were due to mutation of *trpm*, as we observed the same defects using either of two independent *trpm* alleles.

The exacerbation of the *trpm* phenotype by high Mg^2+^ in the food, contrasts with the findings that Mg^2+^ supplementation suppresses the HSH symptoms due to mutations in *TRPM6* [10], [11], the cell death resulting from loss of vertebrate TRPM7 [8] and the growth defect resulting from mutations in the worm *gon-2* and *gtl-1* genes [16]. These effects of high dietary Mg^2+^ are the consequence of the requirement for these other TRPM channels for intestinal and cellular absorption of Mg^2+^. In contrast, loss of *Drosophila trpm* did not appear to have a major impact on gut magnesium resorption, but predominatly impaired the renal organ and excretory function, thereby leading to a defect in the handling of elevated Mg^2+^, resulting in hypermagnesemia. This role appears to be evolutionarily conserved, given the recent observation that the function of the excretory cell in worms in Mg^2+^ removal depends on the TRPM-related channel, GTL-2 [17].

One of the salient defects exhibited by the *trpm* mutants was growth arrest. We suggest that this phenotype was a consequence of reduced anabolism, which was a secondary consequence of the perturbation in Mg^2+^ homeostasis. The problem in anabolism was evident by the smaller size of the cells in fat bodies and the reduced overall protein content.

**Figure 6:**
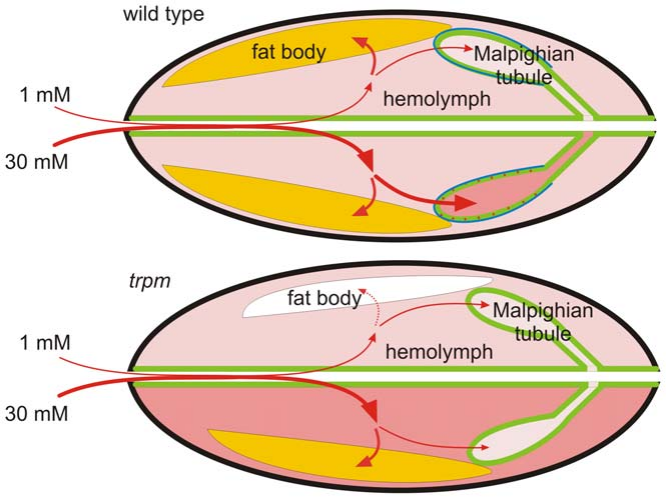
Schematic showing the changes in Mg^2+^ homeostasis in *trpm* mutant larvae. The upper and lower halves of the wild-type and *trpm* larvae depict the main net transport paths for Mg^2+^ upon feeding on a diet containing 1 mM or 30 mM Mg^2+^. The thicker red lines and the darker hue of the background redness over the hemolymph space and the Malpighian tubules represent the higher Mg^2+^ concentration/density in their respective compartments. The white fat body indicates low synthetic capacity. The blue outlines on the initial and transitional segments of the wild-type Malpighian tubules delineate the localization of the TRPM protein.

While high Mg^2+^ largely enhanced the severity of the *trpm* larval growth phenotype, it appeared that supplementation of Mg^2+^ exclusively during the late-stage of larval development partially suppressed the biosynthetic defects in the fat body. In support of this conclusion, the protein content was much higher in larvae fed high Mg^2+^ on day 3 only, than in larvae maintained continuously on food with normal levels of Mg^2+^. These larvae, in contrast to larvae kept continuously on low Mg^2+^, displayed nearly normal hemolymph proteins. We propose that during early larval development when most larval growth is taking place, high Mg^2+^ in the diet is deleterious to *trpm* larvae because the hypermagnesemia suppresses feeding behavior and growth of tissues such as the fat bodies, which is a prerequisite for anabolic function. However, once morphogenesis and tissue growth is largely complete, as in late stage larvae, the high Mg^2+^ may contribute to Mg^2+^ resorption in tissues such as the fat bodies (Figure 6), thereby promoting biosynthetic function.

In summary, we found that the single fly TRPM homolog is an important regulator of Mg^2+^ homeostasis. Loss of fly TRPM impairs renal absorption and excretion of Mg^2+^, thereby resulting in defects in peripheral tissues. This represents the first requirement for a *Drosophila* TRP channel for Mg^2+^ homeostasis, growth and viability. The observation that the sole *Drosophila* TRPM channel functions in Mg^2+^ excretion underscores the broad evolutionarily conserved roles of TRPM channels in Mg^2+^ homeostasis.

## Materials and Methods

### *Drosophila* stocks and maintenance

The following stocks were obtained from the Bloomington Stock Center: 1) w*; In(2LR)noc[4L]Sco[rv9R], b[1]/CyO, P{w[+mC] = ActGFP}JMR1, 2) Df(2R)XTE-18/CyO, 3) *y^1^ w^67c23^*; P[*EPgy2*]*CG34123^EY01618^*/CyO, 4) y^1^ w*; P[*70FLP*]23 P[*70I-SceI*]4A/TM6, and 5) Sp/SM5; Δ2-3, Sb/TM6, *Ubx*.

The flies were maintained on cornmeal-yeast agar medium at 25°C and 70% air humidity on a 12 h light/12 h dark cycle. For analyses of larvae, the adults were pre-fed for 2 days on yeast paste, and embryos were collected on grape juice agar for 3 h-intervals in the dark. Freshly emerged larvae were collected after 18–24 hrs, and kept at 25°C in the dark on agar containing 25% grape juice (diluted in 70 mM KCl, 10 mM NaCl, 30 mM glucose) and dry yeast. The *trpm* mutant larvae were genotyped and separated in advance using a CyO balancer chromosome tagged with Actin5C-eGFP transgene (stock 1, above) [25].

### PCR and Northern blotting

PCR amplification of genomic DNA and cDNAs, which were >3 kb, were performed using the Expand Long Template™. All other PCRs were performed using the Expand High Fidelity™ polymerase kit (Roche Diagnostics). For 5′-RACE, we used the GeneRacer™ RNA ligase mediated RACE kit (Invitrogen). RNA was reverse transcribed using a *trpm*-specific RT primer and a set of staggered PCR primers (*trpm*-RACE 1, 5′ GTCCCACTTTCAAAGGTCTTCTGTATG-3′; RACE-nested 1, 5′-GCCATTGTCGACCAGCAGAAAGTA-3′; -RACE-nested 2, 5′- CGGTTGTGGCCCAGCAGCTCGTGATTG-3′ and RACE-nested 4, 5′-GGATGAGCGCCGCCCTGGAACT-3′) for two rounds of nested PCR against the anchor primers provided with the GeneRacer kit. Semi-nested RT-PCR of the entire *trpm* coding sequence was performed using the RT 1 primer (5′-TTCAACAACTTATTTATCACC-3′) and two rounds of PCR using the 5′ –terminal primers MYF1 (exon 1a, 5′-CGCTTCGATATCGGCTACACC-3′) or MLK1 (exon 1b, 5′-ACCTGTGCCCGTAAAGAAGCCTGTCAAG-3′), which recognize the two alternative start exons, against the 3′ terminal primer TMC (5′-GATCCATTTTCGTCGGCCTTCCACTTT-3′) (30 cycles.) The second PCR step of 35 cycles was carried out after a 1∶50 dilution using the nested oligonucleotides MYF2 (5′-TAATTTTTGGAGCATGTGGATACCC-3′) and MLK2 (5′-CACCACCCAATTTTCCCACTCCTGTTTTC-3′) against TMC, respectively. Reactions were treated for 30 min with Taq polymerase, fractionated on an agarose gel, the bands were excised, gel-purified, and subcloned into a TOPOcloning™ vector (Invitrogen). Single clones were analyzed by digestion with restriction endonucleases to detect length polymorphisms, which might be indicative of alternative mRNA isoforms.

To probe *trpm* RNAs on Northern blots, we isolated total RNA using the TRIzol™ agent (Invitrogen), and purified polyadenylated RNA using the OligoTex™ kit (Qiagen). 1 µg of each polyadenylated RNA was fractionated by electrophoresis on denaturing gels, and transferred to nitrocellulose membranes by capillary action. To prepare the *trpm* probe, we digested pcDNA3-*trpm-K1* with SpeI/XbaI, which excised the entire coding sequence. We then purified the ∼6 kb fragment, and fragmented it with a combination of AgeI/EcoRI/HindIII. The probe was random-labeled using ^32^P-dATP and the Klenow fragment using the HexaLabel™ kit (Fermentas Life Sciences), and purified on a Micro Bio-Spin 6 column (BioRad). The blot was probed overnight in a 0.5 M sodium phosphate buffer (pH 7.0) with 1% BSA, 4% SDS and 3.5×10^6^ cpm/ml at 65°C, washed in 0.1x SSC and 0.1% SDS at 50°C and re-probed with a *Drosophila* α-tubulin 84D probe and washed.

### Generation of *trpm* alleles

We generated the *trpm^1^* allele by imprecise excision of the P-element insertion P[EY01618] (stock 3, see above) using a genetically encoded source of transposase (Δ2-3; stock 5). DNA was prepared from flies that lost the *w^+^* marker encoded in the P-element, placed in pools, and screened by PCR for deletions within the 1.4 kb region encompassing exons C9-C11 using the following primers: *trpm*-excfw (5′-AAGGGGCCCCAATGAGTTTAAGAGACAC-3′) and *trpm*-excrev (5′-GCACACGCAGCACACGCAAAAAGAGTAA-3′). One pool containing a shortened PCR product was characterized further by DNA sequencing (deleted exons C9 – C11 encoding residues 364 to 511).

To generate the *trpm^2^* allele missing exons C2-C4, we used ends-out homologous recombination. To produce the targeting construct, we PCR amplified two *trpm* genomic regions from *w^1118^* adult flies (5′ end of C1 exon defined as nucleotide 1: left arm, 4937 kb, nucleotides −3600 to 1337; right arm, 5022 kb, nucleotides 2980 to 8001), which we subcloned into a modified version of the p[w25] targeting plasmid [18]. We introduced the targeting construct into *w^1118^* flies by germline transformation, crossed transgenic females containing the donor construct inserted on the X chromosome with males carrying transgene insertions on the 3^rd^ chromosome that express the *FLP* and *I-SceI* under control of the *hsp70* promoter (stock 4). F1 females were heat shocked at 37°C for 2 h and mass-crossed to *w^1118^*;Sco/CyO males. Lines in which the donor transgene moved to the 2^nd^ chromosome (*trpm* gene maps to 51E11-51F2) were identified and analyzed by PCR (see Figures 1B and C). We identified one successful targeting event among ∼1000 lines screened.

### Determination of protein levels

We used the Lowry method [26] to determine protein levels. Bovine serum albumin (Fraction V, Sigma-Aldrich) was used as a standard. To compensate for the tissue background in the larval homogenates, we subtracted the absorbance of a blank control.

### Immunofluorescence and *in situ* hybridizations

We probed for *trpm* RNA in wild-type embryos by performing *in situ* hybridizations. To prepare the probes, we subcloned a 920 bp SalI fragment (nucleotides 773–1693 of the coding sequence of the *trpm-K1* cDNA into pBluescript and prepared digoxygenin-labeled sense and antisense cRNAs.

TRPM antibodies were raised in a rabbit against two TRPM peptides, and affinity purified. One of the purified antibodies (P74, raised to NH_3_-SNIKSSTESEKDPPFNET-CONH_2_) efficiently detected the TRPM protein overexpressed in tissue culture cells (data not shown) and was used for further experiments. Malpighian tubules microdissected from 3^rd^ instar larvae were fixed for 10 min in 3.7% formaldehyde solution in 150 mM sodium phosphate buffer (PB, pH 7.4), washed for 10 min in PB and for additional 30 min in PB/0.1% Triton X-100 (PBT). The tubules were preincubated for 30 min in PBT/3% goat serum at room temperature and incubated overnight at 4°C with the TRPM antibodies diluted 1∶500 in PBT/goat serum. The samples were washed in PBT and incubated for 2 h at room temperature with a goat anti-rabbit-IgG Alexa 488 conjugate (Molecular Probes/Invitrogen) diluted 1∶2000 in PBT/3% goat serum. After washing and mounting, the samples were analyzed using a Zeiss LSM 510meta confocal laser scanning microscope.

### Determination of Mg^2+^ and Ca^2+^ levels

To determine Mg^2+^ content in larval tissues, we used the azo dye Eriochrome Black T [27]. Larvae were washed for 10 min in deionized water prior to isolating the tissues or bleeding out the hemolymph. To assay Mg^2+^ in the hemolymph using a filter-based assay, we tore open the larvae and allowed the hemolymph to drain onto 3 MM Whatman paper. Whole larvae, bled carcasses or microdissected Malpighian tubules were collected in 100 µl of water, homogenized with a micropestle and sonicated. 1 mM Eriochrome Black T in ethanol was diluted 50-fold in a 50 mM ammonia/10 mM ammonium chloride buffer (pH 10.0). 50 µl of the samples or MgCl_2_ standards (ranging from 50 µM-1 mM) were added to 800 µl dye dilution and absorptions at 530 and 660 nm were measured using an Uvikon 930 spectrophotometer (Kontron Instruments). The 530/660 nm ratios were used to determine the Mg^2+^ concentrations using the calibration curve obtained with the known Mg^2+^ standards.

To determine the hemolymph Mg^2+^ concentration using a solution assay, we adapted a procedure described previously [28]. 8–10 3^rd^ instar larvae were washed for 10 min in deionized water and blotted dry on Whatman 3 MM paper. The larvae were then torn open with clean microforceps and placed on top of a column consisting of a cut 1 ml pipet tip filled with glass wool, which was inserted into a 0.5 ml microcentrifuge tube cooled on ice. The assemblies were centrifuged at 4°C at 600 g for 5 min, the hemolymph samples were collected, diluted 100-fold and the Mg^2+^ levels measured with Eriochrome Black T.

Hemolymph Ca^2+^ levels were determined using the arsenazo III dye [29]. 40 µl of hemolymph (diluted 1∶100) or Ca^2+^ standards were added to 700 µl of 50 µM arsenazo III in 100 mM MES (morpholino ethane sulphonic acid, pH 6.5) in deionized water. The absorptions were quantified at 660 nm and Ca^2+^ concentrations were derived using the standard Ca^2+^ curve.

## Supporting Information

Figure S1 Cloning of trpm cDNAs and developmental expression of the trpm mRNA. (A) Cladogram including TRPM proteins in humans, worms and several insects. The relationships were based on the amino acid sequences encompassing the pore and S6 domains and calculated using the ClustalW algorithm. (B) Alternative *Drosophila trpm* mRNA isoforms. The lengths of the encoded protein isoforms in amino acids (aa) are indicated. (C) Developmental RNA blot hybridized with a trpm DNA probe. The blot contained 1 µg of poly(A)+ RNA prepared from 5–15 h embryos (E1), 16–20 h embryos (E2), the three larval instar stages (L1-L3), prepupal stage (P0), day 2 pupa (P1), mature pupae (day 4–5, P2), female adults (F), male adults (M) and *Drosophila* S2 cells.
(1.50 MB TIF)

Figure S2 Representative fat bodies microdissected from *trpm^2^*, *trpm^2^* heterozygous and wild type (w1118) larvae that were either kept on medium containing 1 mM or 30 mM total magnesium, as indicated. The scale is the same in all panels.
(1.38 MB TIF)
