# Supplementary figures and images for: Drosophila TRPM Channel Is Essential for the Control of Extracellular Magnesium Levels
Source: PLoS One. 2010 May 6;5(5):e10519. doi: 10.1371/journal.pone.0010519 (PMC2865541; doi:10.1371/journal.pone.0010519)

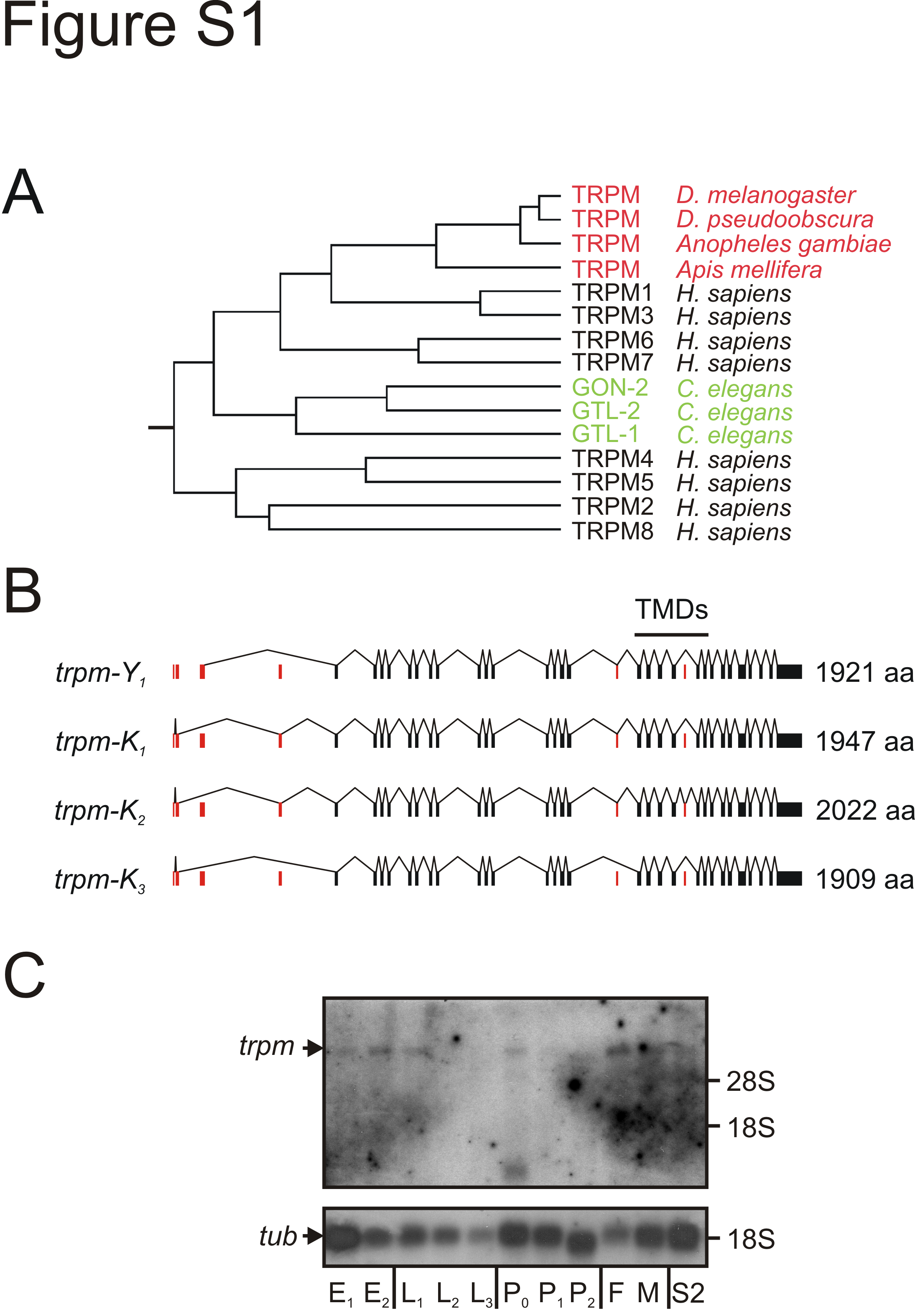

Supplement: Figure S1 — Cloning of trpm cDNAs and developmental expression of the trpm mRNA. (A) Cladogram including TRPM proteins in humans, worms and several insects. The relationships were based on the amino acid sequences encompassing the pore and S6 domains and calculated using the ClustalW algorithm. (B) Alternative Drosophila trpm mRNA isoforms. The lengths of the encoded protein isoforms in amino acids (aa) are indicated. (C) Developmental RNA blot hybridized with a trpm DNA probe. The blot contained 1 µg of poly(A)+ RNA prepared from 5–15 h embryos (E1), 16–20 h embryos (E2), the three larval instar stages (L1-L3), prepupal stage (P0), day 2 pupa (P1), mature pupae (day 4–5, P2), female adults (F), male adults (M) and Drosophila S2 cells. (1.50 MB TIF) [file pone.0010519.s001.tif]

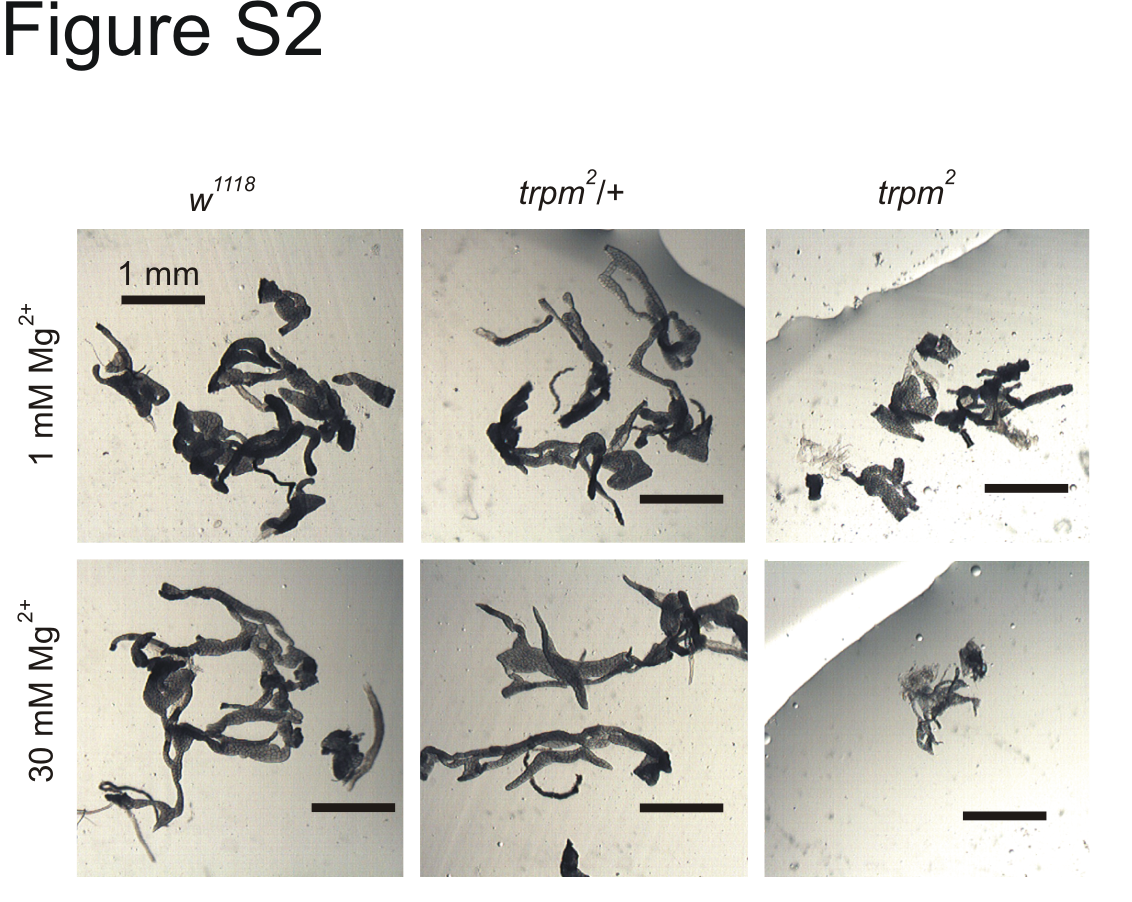

Supplement: Figure S2 — Representative fat bodies microdissected from trpm2, trpm2 heterozygous and wild type (w1118) larvae that were either kept on medium containing 1 mM or 30 mM total magnesium, as indicated. The scale is the same in all panels. (1.38 MB TIF) [file pone.0010519.s002.tif]
